# Study of Structural stability and formation mechanisms in DSPC and DPSM liposomes: A coarse-grained molecular dynamics simulation

**DOI:** 10.1038/s41598-020-58730-z

**Published:** 2020-02-04

**Authors:** H. Hashemzadeh, H. Javadi, M. H. Darvishi

**Affiliations:** 1 Nanobiotechnology Department, Faculty of Bioscience, Tarbiat Modares University, Tehran, Iran; 2 Nanobiotechnology Research Center, Baqiyatallah University of Medical Sciences, Tehran, Iran

**Keywords:** Computational models, Nanostructures, Computational biophysics, Molecular self-assembly, Computational science

## Abstract

Liposomes or biological vesicles can be created from cholesterol, phospholipid, and water. Their stability is affected by their phospholipid composition which can influence disease treatment and drug delivery efficacy. In this study, the effect of phospholipid type on the formation and stability of liposomes using coarse-grained molecular dynamics simulations is investigated. For this purpose, the simulation study of the DSPC (1,2-distearoyl-sn-glycero-3-phosphocholine) and DPSM (Egg sphingomyelin) lipids were considered. All simulations were carried out using the Gromacs software and Martini force field 2.2. Energy minimization (3000 steps) model, equilibrium at constant volume to adjust the temperature at 400 Kelvin and equilibrium at constant pressure to adjust the pressure, at atmospheric pressure (1 bar) have been validated. Microsecond simulations, as well as formation analysis including density, radial distribution function, and solvent accessible surface area, demonstrated spherical nanodisc structures for the DPSM and DSPC liposomes. The results revealed that due to the cylindrical geometric structure and small-size head group, the DSPC lipid maintained its perfectly spherical structure. However, the DPSM lipid showed a conical geometric structure with larger head group than other lipids, which allows the liposome to form a micelle structure. Although the DSPC and DPSM lipids used in the laboratory tests exhibit liposome and micelle behaviors, the simulation results revealed their nanodisc structures. Energy analysis including overall energy, Van der Waals interaction energy, and electrostatic interaction energy showed that DPSM liposome is more stable than DSPC liposome.

## Introduction

Liposomes, or small vesicles, can be created from non-toxic (normal) cholesterol and phospholipids. Liposomes were introduced in 1965 and initially used as models for studying biological membranes^1^. Later, Liposomes have been considered as drug delivery systems. Solutions of phospholipids in water exhibit a large variety of aggregation states such as double layer or liposome membrane^2,3^. Characteristics such as inherent low toxicity, biodegradability and lack of immunogenicity qualify Liposomes as a considerable carrier in novel drug delivery systems^4–7^. In addition, Liposomes are dynamic, so different structures can be achieved depending on the phospholipids types^8,9^. It is now possible to engineer a wide variety of sizes, phospholipids, and liposomal superficial properties^10^.

The liposomal surface can be engineered and functionalized by selecting special lipids, in facilitating covalent binding of proteins (e.g. antibodies and proteins to sugars, *i.e* lectin), glycoproteins and synthetic proteins^11^. Liposomes are widely used in vaccines, enzymes and drug (insulin and anticancer drugs) carriers^12^. In general, the highly unsaturated phospholipid compounds can lead to the instability of the liposome structure^13^. Lipids derived from biological sources such as eggs and soybeans typically consist of significant levels of unsaturated fatty acids, thus inherently are less stable than their counterparts. While saturated lipids are more stable, they have a higher transition temperature^14^. Liposomes containing saturated phospholipids showed increased stability and high transition temperature compared to liposomes composed of unsaturated phospholipids. Hence, to liposome synthesizing purposes if unsaturated phospholipids is essential, it is important that keep the transition temperature degree as low as possible. The existence of Polyethylene Glycol (PEG) on the surface of liposomal delivery systems has shown to increase the half-life in blood-circulation^15^, while reducing toxicity and exposure of healthy cells to drug toxicity, i.e. drugs in vulnerable tissues such as the liver and kidneys^16–19^. In addition, the combination of PEG with liposome has resulted in improvement of liposomal stability^15,20^. The most important obstacle in liposomal technology is their long-term instability, especially when used as drug carriers^21^. Physical and chemical stability of liposomes are affected by various factors influencing the liposomes stability and the effectiveness of drug penetration^22^. For this reason, the stability of liposomes is critical for long time circulation. Long-term stability of liposomes containing pharmaceutical substances is strongly influenced by the type of phospholipid’s liposome structure.

The main advantage of molecular dynamic simulations is obviously the decreased costs^23^. Molecular dynamics simulation is useful tools that offer information about biomolecular hydrodynamic behavior^9,15^. In other words, fundamental understandings about stability and formation mechanisms of lipids especially liposomes could provide a guideline for rational design of them. Molecular dynamics simulation is helpful to open the new opportunities to further investigate liposomes structure and functionality. Coarse-grained (CG) models offer simulation of larger systems like lipids for longer times by decreasing the number of degrees of freedom (df) compared with all-atom models^23^.

**Figure 1:**
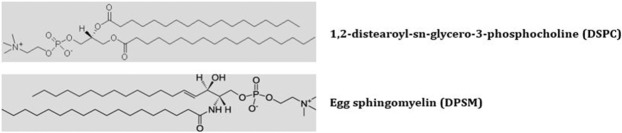
1,2-distearoyl-sn-glycero-3-phosphocholine and Egg sphingomyelin phospholipids.

In this research, the effect of phospholipid type on the formation and stability of liposomes using coarse-grained molecular simulations is studied. For this purpose, the liposomes of DSPC (1,2-distearoyl-sn-glycero-3-phosphocholine) and DPSM (Egg sphingomyelin) were simulated. Figure 1 shows the two types of phospholipid used in this study received from the cgmartini and Avantilipids webpages. DSPC and DPSM phospholipids are issued in the laboratory to synthesize spherical liposomes. The DSPC phospholipid creates spherical liposomal structures, while DPSM phospholipid develops a double layer membrane structure. Coarse-grained simulations consider similar atoms close together as a sphere, and allow simulations of systems that are not available at all common atomic time scales^24,25^.

## Results and Discussion

**Figure 2:**
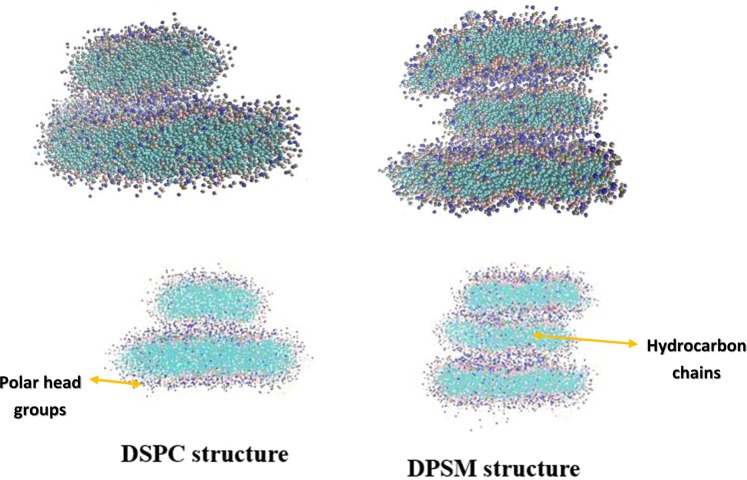
The final structure of lipids of DSPC and DPSM after 1000 nanoseconds (1 microsecond).

Today, many theoretical and empirical studies investigate the formation and stability of liposomes, due to their importance as drug carriers, and are experimentally synthesized in laboratories. However, molecular dynamics simulation has the ability to deliver more detailed information in the field of biosynthesis and drug delivering with regards to the high-cost of materials used for liposomes preparations, as well as limited documentation on stability of liposomes and its molecular arrangement^26^. In fact, molecular dynamics simulation, as a primary study approach, helped to better investigate the liposomes formation process and stability. Using molecular dynamics simulation, the effect of different factors on the formation of liposomes and their stability was examined, so that an ideal liposome can be synthesized. In addition, the role and contribution of each interaction in the liposomes formation and stability can be evaluated. DSPC and DPSM Liposomes are experimentally synthesized and are the most important carriers as drug delivery system, including anticancer drugs. In this research, the main goal was to study the stability parameters and the DSPC and DPSM liposomes formation. The effective parameters in formulation and stability were extracted using data obtained from the simulation path file. The results showed differences in the formation and stability of DSPC and DPSM liposomes. In this study, in the self-assembly process, when the lipids are uniform and homogenously distributed in water, the formation rate of liposomes is faster than when the lipids had bicelle clumps and other heterogeneous structures. The same results were reported by Chng *et al*. They examined the temperature effect on accelerating the formation of vesicles by DPPC lipid molecules caused by different water molecules per amphiphilic ratios. They concluded the binary associations between micelle-like lipids^27^. Figure 2 shows the final structure of DSPC and DPSM liposomes after 1000 nanoseconds (1 microsecond). The results show that the DSPC lipid has two nanodisc structures and the DPSM lipid has three nanodisc structures in the simulation environment. The DSPC lipid has a cylindrical geometric structure and a small-sized head group that forms the spherical liposomal structure. On the other hand, the DPSM lipid has a conical geometric structure with a larger polar head group than another lipid, which allows the liposome to form a micelle structure. Although the DSPC and DPSM lipids used in the laboratory are always in liposome and micelle forms, they developed nanodisc structures in the simulation process^28^.

### The formation of liposomes

**Figure 3:**
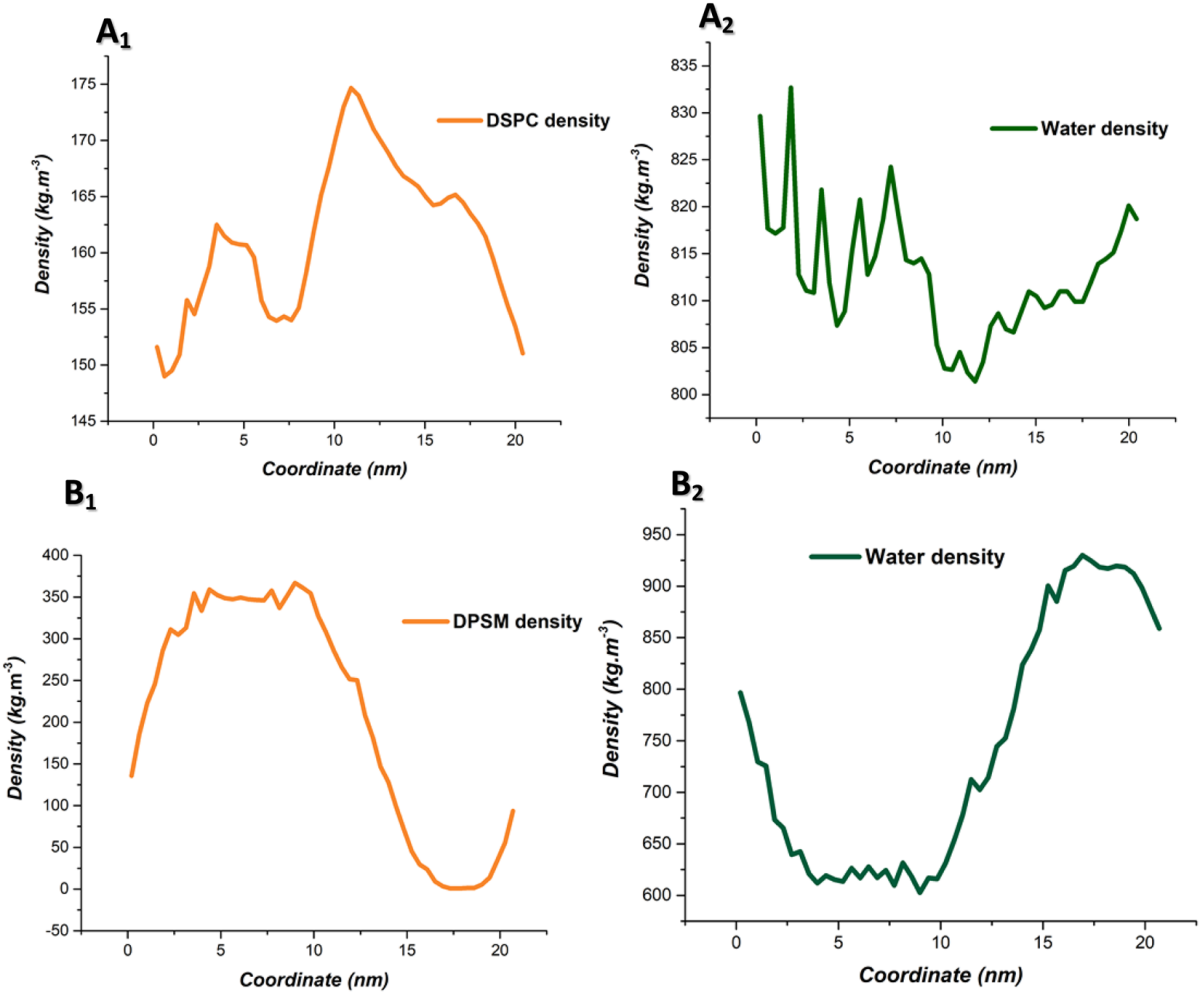
Density analysis shows that nanodisc structures have been created after simulation time. **(A1**) density of DSPC lipid molecules (**A2**) density of water molecules (**B1**) density of DPSM lipid molecules (**B2**) density of water molecules.

**Figure 4:**
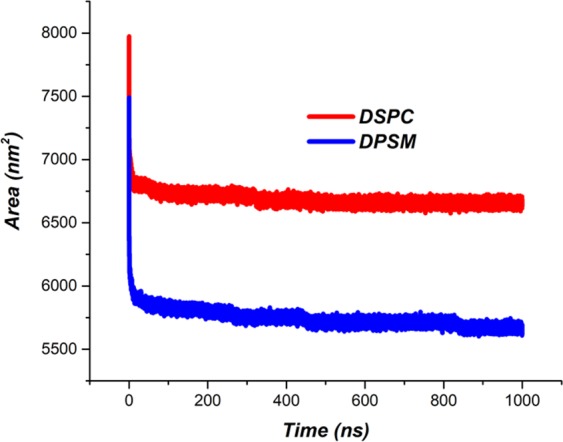
Solvent accessible surface area analysis for DSPC and DPSM liposome. The graph shows the amount of solvent.

In this study, density, gyration radius, Radial Distribution Function (RDF), Root Mean Square Deviation (RMSD) and Solvent Accessible Surface Area (SASA) were employed to verify the liposomes formation^29,30^. Using density analysis, the density profiles of lipid and water molecules were calculated along the Z direction. The density results reveal a nanodisc structures in the final stage^31,32^. Figure 3(A1,A2,B1,B2) shows the density of lipid and water molecules. Figure 3(A1,B1) and Fig. 3(A2,B2) show the density of lipids and water molecules, respectively. The results reflect differences between lipid density profiles and water density in DSPC and DPSM lipids. According to Fig. 4B, the density of water molecules is low at the exact regions where the density of lipid molecules is high. The DSPC density profile shows a sharp peak in comparison with the DPSM lipid one, which could be related to different structure formations during self-assembly. Based on the results (Fig. 2), DSPC and DPSM are asymmetric and the lipids show an ordered cylindrical and spherical geometric structure.

**Figure 5:**
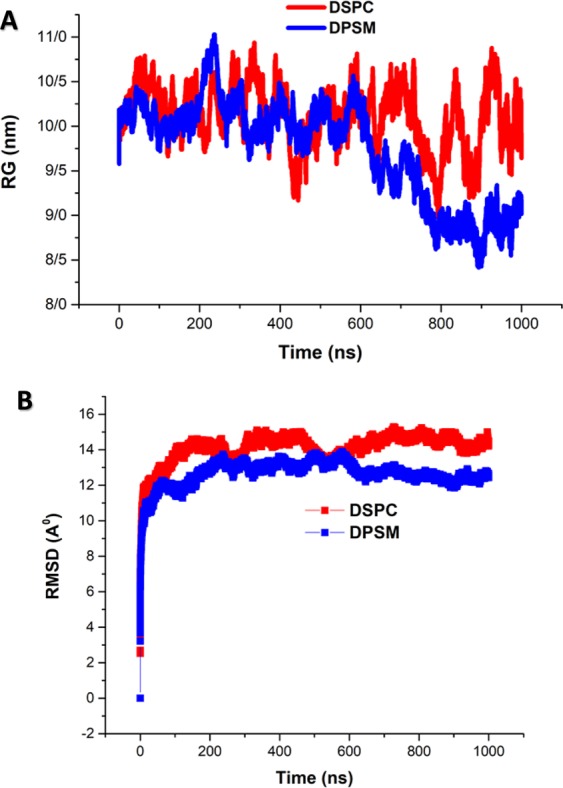
Radius of gyration and RMSD analysis during the simulation process.

In order to evaluate structural compression changes, the gyration radius diagram of each structure was recorded during the simulation time. The initial value of Rg for the DSPC and DPSM structures were 9.6 and 9.8 nm respectively. After completing the simulation and formation of nanodisc structures, analysis of the gyration radius of the total lipids was obtained. As shown in Fig. 5A, the analysis of the gyration radius shows that the DSPC and DPSM liposomes are compressed throughout the simulation time^33,34^. In MD simulation, the gyration radius is an index to monitor the structural formation process^35^. As shown in Fig. 5A, a sudden drop occurred in the gyration radius profile of the DSPC lipid which indicates structural transformation, while sharp and sudden drops and peaks are not observed in the DSPM lipids^26^. The RMSD for each structure is based on its previous structure. Figure 5(B) shows the analysis of the RMSD parameter for the simulated lipid structures. As shown in Fig. 5(B), it is evident that the time required for attaining stable DSPC and DPSM structural formations is around 1 nanosecond, after which the RMSD reached a constant value after a few nanoseconds^36^. Valeriya M. Trusova and *et al*., reported a steady increase of RMSD value during the first 22 ns, and fluctuations were observed around an average value (i.e. ~0.21 ns). They concluded that the backbone RMSD value decreases due to partial unfolding. Moreover, lysozyme intramolecular leads to RMSD changes^37^.

**Figure 6:**
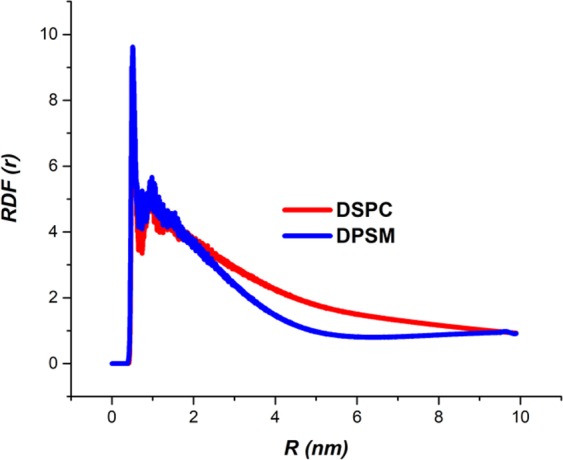
Radial distribution function analysis of DSPC and DPSM lipids. In both lipids, the source distance between the two side lipids is considered 0.5 nm.

A Radial Distribution Function (RDF) is applied to describe the average structure of dynamic molecules and is a basic method for measuring the structure of a dense material^38^. The RDF results show that DSPC and DPSM structures have a dense structure (Fig. 6). For better characterization of head group interaction, the RDF between the nitrogen (N4) in the choline and the phosphates (P8) at the two side of neighboring lipids in the head groups (DPSM and DSPC liposomes) were calculated^39^. For calculation of the RDF in both liposomes, the source distance between the lipids was considered equal to 0.5 nm^40^. The developed nanodiscs had an homogeneous structure and as a result, the RDF value increased at a certain distance (0.5 nm), followed by a significant reduction^41^. The distance between phosphate and nitrogen particles in the headgroups of DSPC and DPSM lipid molecules in a nanodisc display a sharp peak. The peak locations of the lipids headgroups correspond to the inner and outer radius of the nanodisc (see Fig. 6)^42^. It is likely that the DSPC and DPSM lipids are sufficiently equilibrated on such long time-scales (1000 ns). Choon-Peng Chng obtained partial equilibrated structures for monolayer lipids, suggesting longer simulation time to facilitate the flip-flops, as no flip-flops occurred during their simulations^27^.

Solvent Accessible Surface Area (SASA) analysis was performed to verify the membrane and liposomal structural formation. Results from previous studies show that the amount of SASA is reduced by the formation of liposomes and membrane structures^43^. As shown in Fig. 4, when the nanodiscs are formed, water penetration into the structures is prevented as a result. The results show that SASA values for the DSPC structure was higher than that of the DPSM structure (Fig. 4). The DSPC lipid has a low phase transition temperature and being more fluid at 400 °C, in turn leads to high SASA values. This issue reflects as increase in fluidity of the DSPC lipid and the weakening of the lipid packing as well as an increase in the space among them. In addition, analyzing the SASA is also used in investigation efforts on molecular structure and dynamics^44–46^.

### Energy analysis

Energy analyses (overall energy, Van der Waals and Electrostatic energies) was carried out to evaluate the energies and interactions contributing to the stability of the formed structures^47^. To obtain the final stability state of liposomal structures, various energy analyses were conducted. The results of all energy analyses indicate the stability of the developed structures. According to the energy diagram, a decreasing system energy was observed due to the liposomal system formation during the simulation^48^. Energy analysis was also performed at the equilibrium stage to check the stability of the system at this stage^49^.

### The effects of Van der Waals and Electrostatic energies on the stability of DSPC and DPSM liposomes

**Figure 7:**
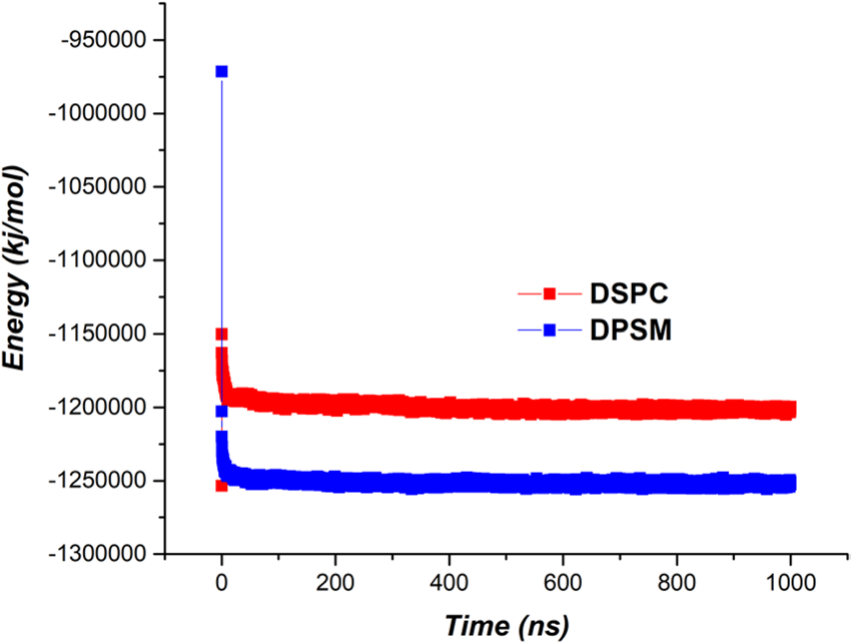
The energy of Van der Waals interactions for DPSM and DSPC liposomes.

As shown in Fig. 7, the Van der Waals interactions were calculated for the liposomes. At the beginning of the simulation, the Van der Waals energy was low, but with the formation of Van der Waals interactions, its amount increased (became negative). The result showed that Van der Waals energy levels for the DPSM liposome are higher than that of the DSPC liposome, indicating greater amounts of Van der Waals interactions is in this liposome. The nanodisc structure of the DPSM liposome makes it possible to produce more Van der Waals interactions^50^.

**Figure 8:**
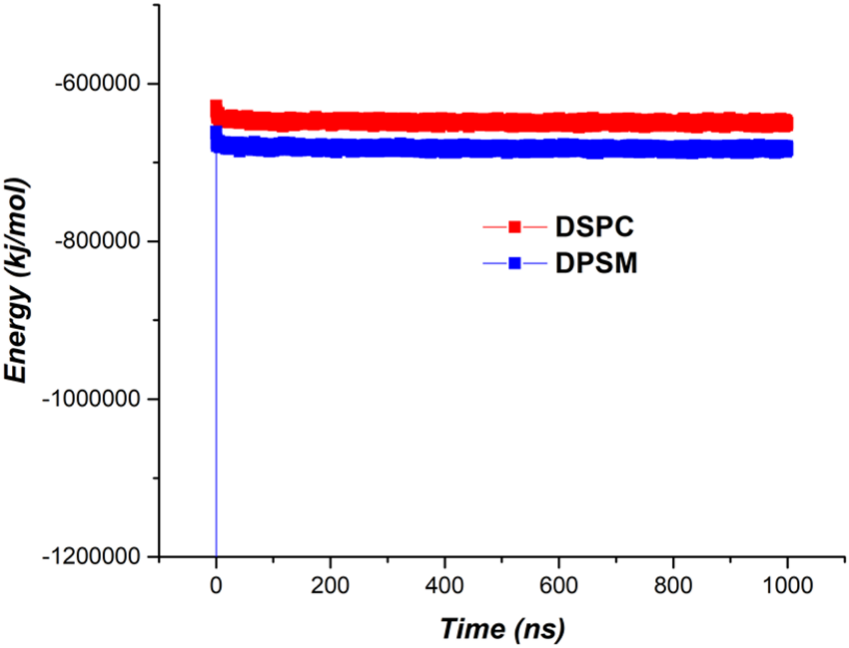
The energy of Electrostatic interactions for DPSM and DSPC liposomes.

Electrostatic interactions for both liposomes are presented in Fig. 8. It can be seen that Electrostatic energy levels for the DPSM liposome is higher than the DSPC liposome, indicating the greater number of Electrostatic interactions.

### Study of stability of DSPC and DPSM liposomes using overall energy analysis

**Figure 9:**
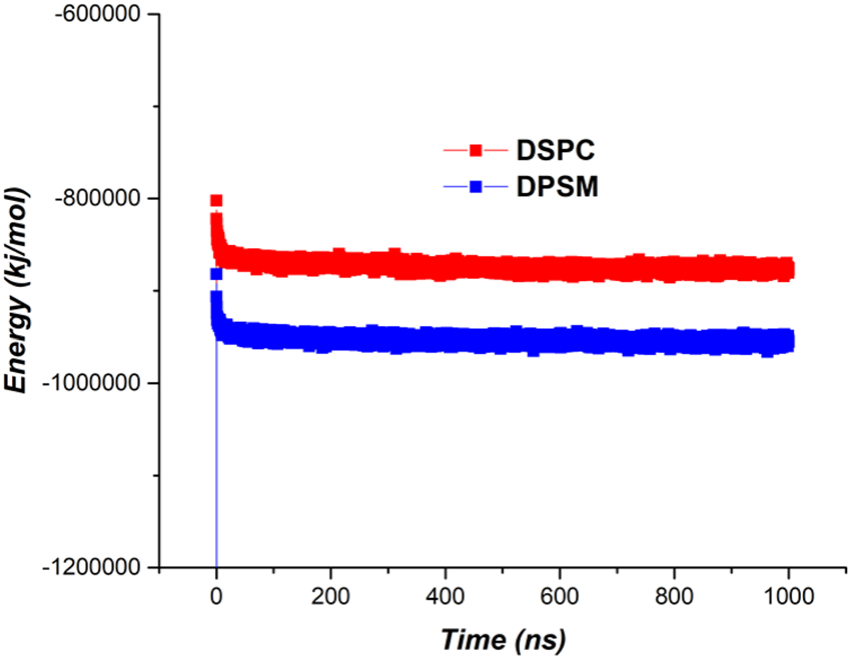
Overall energy of DPSM and DSPC liposomes.

The final stability analysis of the developed structures was calculated using overall energy analysis. As shown in Figs. 7 and 8, the amount of Van der Waals and electrostatic energy for the DPSM Liposome are greater than that of the DSPC liposome. In Fig. 9, it is evident that the overall energy of the DPSM liposome is greater than that of the DSPC liposome that have developed the nanodisc structure. As a result, the DPSM liposome is more stable than the DSPC liposome^51–53^.

## Conclusions

Liposomes carriers are widely used in biology and medicine and are implemented as carriers for many medications and biological molecules delivery. To reach high performances of the liposomes, they should be well-formed. Today, many theoretical and experimental studies investigate liposomes formation and stability. Molecular dynamics simulation, as a method of quantitative mathematical principles, is a complementary method to study the macromolecules mechanisms and dynamics, and biological phenomena. In this study, two types of liposomes were simulated to investigate their formation and stability. Self-assembly approaches of two types of phospholipid, namely, DSPC and DPSM, from random initial configuration was simulated. The MARTINI coarse-grain model and GROMACS package was employed in this study. The simulations reveal that there is one basic self-assembled structure, i.e. the nanodisc structure. Formation analyses, including density, radial distribution function, solvent accessible surface area, gyration radius, and root mean square deviation were applied. The formation analyses showed that the DSPC lipid has two structures of nanodisc while the DPSM lipids have three nanodisc structures in the simulation environment. It seems that the physicochemical properties have a pivotal role in the structure of each liposome in terms of structural formation and stability. The DSPC lipid had a cylindrical geometric structure and a small-sized head group originally, that forms the bilayer structure. Meanwhile, the DPSM lipid had a conical geometric structure and has also a larger head group than other lipids, which allows the liposome to form a micelle structure. Although DSPC and DPSM lipids are used in the laboratory and produce liposome and micelle, they have developed nanodisc structures in this study. In addition, to check the stability of the developed structures, energy analyses including Van der Waals interactions, electrostatic interactions, and overall energy interactions were performed. All the energy analyses indicate that the DPSM liposome was more stable than the DSPC liposome. RMSD and energy profile results for these structures indicated their stability. Bilayer, micelles, hexagonal structures and etc., can be formed during the simulation process of lipids. The results of energy profile analysis confirmed this hypothesis that the nanodisc structure is more stable than other formation. Regarding the timescales of the simulations presented in this study (1000 ns), headgroup-headgroup binding of lipids improved the Van der Waals and Electrostatic interactions significantly.

Arnarez *et al*.^23^, introduced a novel Coarse-Grained Martini model, “Dry” Martini. They showed that, their model is able to study the lipid membrane properties including bilayer thickness, area per lipid, bending modulus, and coexistence of liquid-ordered and disordered domains. Moreover, they showed that the Dry Martini can be used to study membrane fusion and formation of tether, with results similar to those of the standard Martini model. By this model, they noted that the membrane proteins could also be investigated, but less quantitative results they obtained. In comparison with current study, one of the most important aspect of MD simulation is speedups for large systems. Arnarez *et al*., in their study concluded that the absence of water in Dry Martini leads to a significant speedup for large systems, and suggested the new model is able to the study of complex multicomponent membranes containing millions of lipids. The results of this study provided significant evidence for the importance of the molecular dynamics simulations on the structural stability and mechanisms of formation of DSPC and DPSM lipids, these can be useful as a guide to the experimental synthesis of these lipids. The results of this simulations study revealed that, bilayer nanodisc structures are stable than others structures, which could help to design of lipids like liposome with high efficacy for pharmaceutical applications.

## Materials and Methods

**Figure 10:**
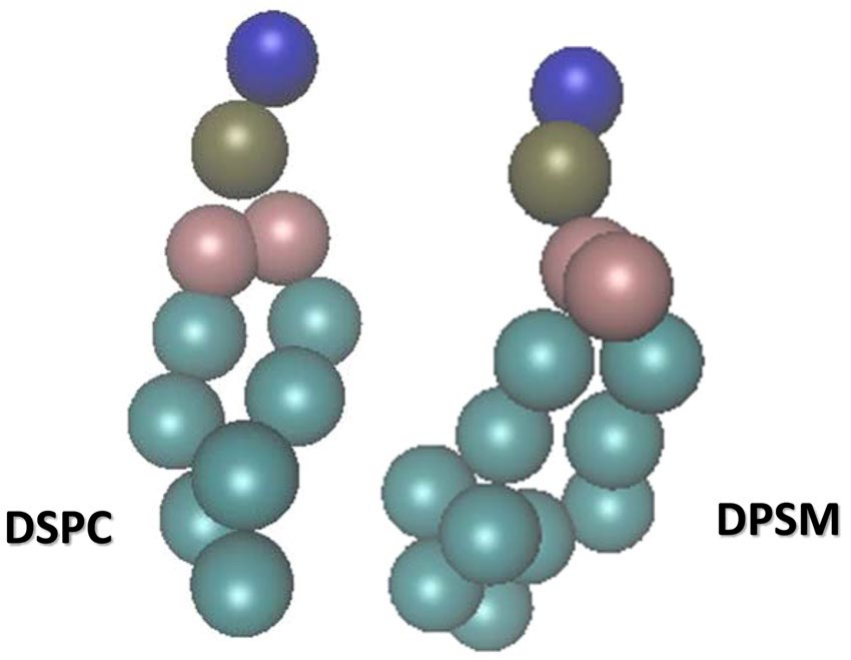
The coarse grain structure of 1,2-distearoyl-sn-glycero-3-phosphocholine and Egg sphingomyelin phospholipids obtained from CHARMM site.

In this research, the formation and stability of the phospholipids were investigated in the simulation environment^54^. The time scale was considered at the microsecond. In the martini model, there are four types of atoms including Apolar (C), Polar (P), Non-polar (N) and Charged (Q)^55^. DSPC phospholipids includes grains of NC31 (blue color), PO4 (gray), GL1 and GL2 (pink), C1Am, C2A, C3A, C4A, C5A, C1B, C2B, C3B, C4B and C5B (turquoise blue). And Dpsm phospholipids includes grains of NC3 (blue), PO4 (gray), AM1 and AM2 (pink), T1A, C2A, C3A, C1B, C2B, C3B and C4B (turquoise color). The initial data of the phospholipid components (including phospholipids of 1,2-distearoyl-sn-glycero-3-phosphocholine and Egg sphingomyelin) were obtained as a coarse grain state from the web server CHARMM-gui (http://www.charmm-gui.org/) (Fig. 10). CHARMM-gui was used for the construction of initial structures of the phospholipids and then simulations were carried out using the Gromacs software (version 5.0.1)^56^. The main parameter files (*i.e*. ITP and TOP files), which are required to initiate the simulation process in the Gromacs environment, was taken from the CHARMM-gui website^26,57^.

### Creating the boxes and hydration of phospholipids

**Figure 11:**
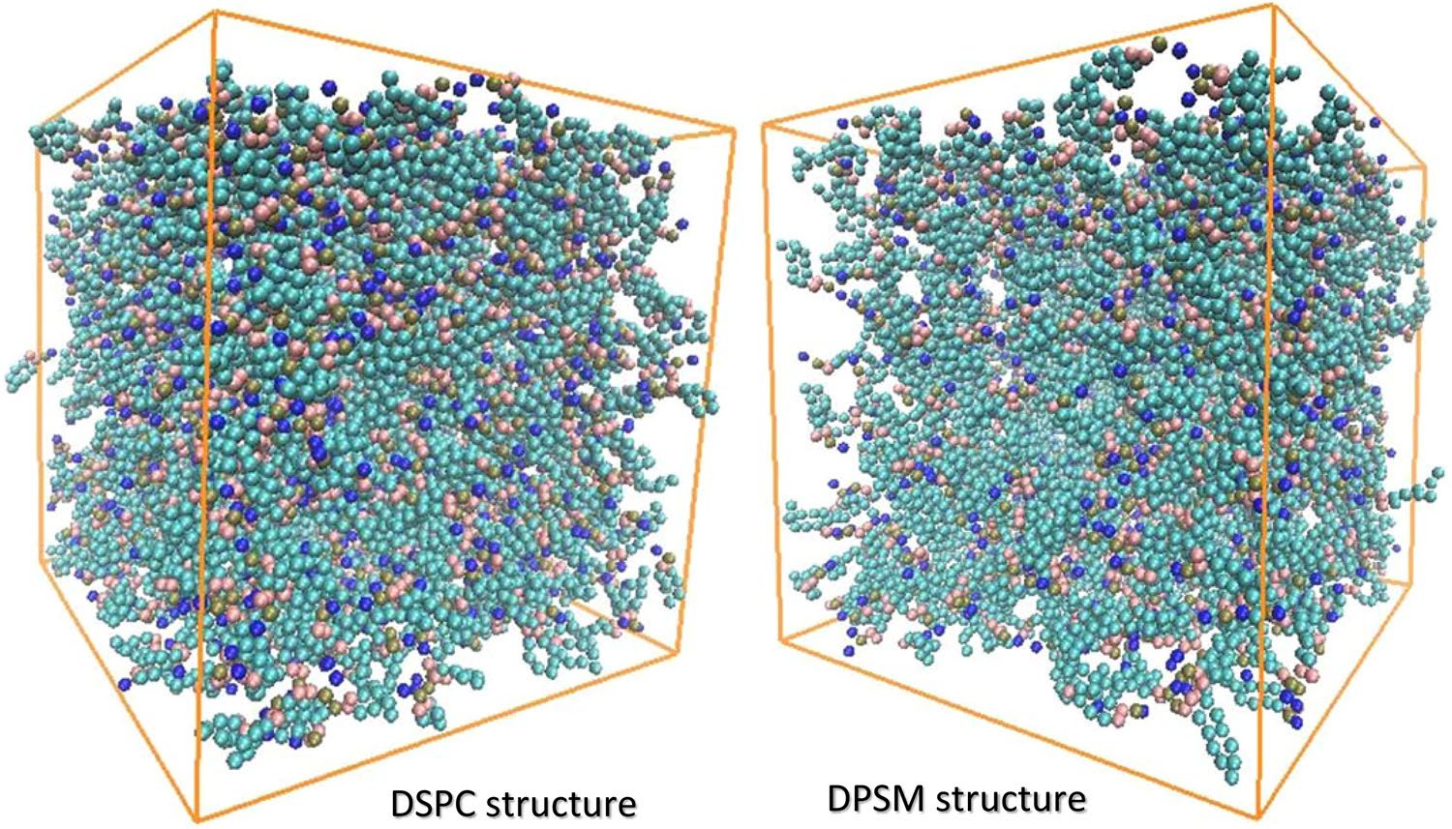
Simulation box and initial configuration defined for DSPC and DPSM lipids. For the DSPC liposome, 901 molecules 1,2-distearoyl-sn-glycero-3-phosphocholine and 60311 molecules of water, and for the DPSM liposome, 901 molecules of Egg sphingomyelin and 60311 molecules of water were placed in the box.

In this study, each phospholipid molecule was placed randomly in a cube-shaped box of 20 nm dimensions. A certain number of phospholipid molecules was added to each of the box systems (Fig. 11). For the DSPC liposome, 901 molecules 1,2-distearoyl-sn-glycero-3-phosphocholine and 60311 molecules of water, and for the DPSM liposome, 901 molecules of Egg sphingomyelin and 60311 molecules of water were placed in the box. The parameters used in this study were provided based on the proportions and concentration results from previous research works, reported for the liposome synthesization. Water molecules were added to the system for watering. The type of water used in this research was coarse-grain and polarized Martini model^22,58–60^.

### Molecular dynamics simulation studies

In this study, two molecular dynamics simulation processes were performed for DSPC and DPSM liposomes. The simulation time for each liposome was 1,000 nanoseconds (1 microsecond). All simulations were performed using the Gromacs software and Martini force field 2.2.^61–63^. Energy minimization steps were equal to 3000 steps and equilibrium in constant volume to adjust the temperature at 400 Kelvin and equilibrium at constant pressure to adjust the pressure, at atmospheric pressure (1 bar). Besides, the time step was set 20 femtoseconds^64–66^.

The mdp file entries were also set. The nst list parameter was set equal to 10. The coulomb-type and vdw_type parameters are defined as cut-offs, and the algorithms were respectively defined for the t-coupl, p-coupl and cutoff-scheme parameters including v-rescale, berendsen and verlet.

### Results analysis of molecular dynamics simulation

After completing the MD simulation steps, the results were recorded in the output files (EDR, TPR, XTC, GRO, etc.). The results were used to investigate the formation and stability of liposomal structures, including analysis of density, gyration radius, radial distribution function, and solvent accessible surface area. An overall energy analysis was also carried out.
